# Crystallographic and Computational Characterization of Methyl Tetrel Bonding in S-Adenosylmethionine-Dependent Methyltransferases

**DOI:** 10.3390/molecules23112965

**Published:** 2018-11-13

**Authors:** Raymond C. Trievel, Steve Scheiner

**Affiliations:** 1 Department of Biological Chemistry, University of Michigan, Ann Arbor, MI 48109, USA; 2 Department of Chemistry and Biochemistry, Utah State University, Logan, UT 84322, USA; steve.scheiner@usu.edu

**Keywords:** noncovalent bond, sigma-hole, charge transfer, molecular electrostatic potential, tetrel bond, methylation, methyltransferase, methyl transfer, S-adenosylmethionine, AdoMet, SAM, S_N_2 reaction

## Abstract

Tetrel bonds represent a category of non-bonding interaction wherein an electronegative atom donates a lone pair of electrons into the sigma antibonding orbital of an atom in the carbon group of the periodic table. Prior computational studies have implicated tetrel bonding in the stabilization of a preliminary state that precedes the transition state in S_N_2 reactions, including methyl transfer. Notably, the angles between the tetrel bond donor and acceptor atoms coincide with the prerequisite geometry for the S_N_2 reaction. Prompted by these findings, we surveyed crystal structures of methyltransferases in the Protein Data Bank and discovered multiple instances of carbon tetrel bonding between the methyl group of the substrate S-adenosylmethionine (AdoMet) and electronegative atoms of small molecule inhibitors, ions, and solvent molecules. The majority of these interactions involve oxygen atoms as the Lewis base, with the exception of one structure in which a chlorine atom of an inhibitor functions as the electron donor. Quantum mechanical analyses of a representative subset of the methyltransferase structures from the survey revealed that the calculated interaction energies and spectral properties are consistent with the values for bona fide carbon tetrel bonds. The discovery of methyl tetrel bonding offers new insights into the mechanism underlying the S_N_2 reaction catalyzed by AdoMet-dependent methyltransferases. These findings highlight the potential of exploiting these interactions in developing new methyltransferase inhibitors.

## 1. Introduction

Methyltransferases represent a ubiquitous class of enzymes that methylate a vast array of small molecules and macromolecules and participate in numerous biological processes, including metabolism, signal transduction, and gene expression [1,2,3]. The majority of these enzymes utilize the methyl donor S-adenosylmethionine (AdoMet) whose methyl group is rendered highly reactive through its bonding to a sulfonium cation in the substrate. AdoMet-dependent methyltransferases catalyze an S_N_2 reaction wherein a nucleophilic atom, such as oxygen, nitrogen, or sulfur, attacks the electrophilic methyl carbon atom of AdoMet, with the sulfur atom displaced as the leaving group [4]. The reaction mechanism of these enzymes has been a subject of intense study for over 40 years [5] and has led to the proposal of several different models for catalysis. These models include (1) compression or compaction of nucleophile, electrophile, and leaving groups along the reaction coordinate [6,7,8,9], (2) formation of near attack conformers (NACs) that align the nucleophile and methyl group in a productive geometry for the S_N_2 reaction [10,11,12,13], (3) electrostatic pre-organization within the active site that promotes methyl transfer [14,15], and (4) cratic effects involving the free energy of association of the substrates in a catalytically favorable alignment within the active site [16,17]. Despite these models, the methyltransferase mechanism remains a topic of active debate.

Recent structure–function studies of methyltransferases have explored the interactions between their active sites and the AdoMet sulfonium cation. A survey of high-resolution crystal structures of methyltransferases in the Protein Databank (PDB) identified unconventional carbon–oxygen (CH∙∙∙O) hydrogen bonds between the AdoMet methyl group and oxygen atoms within the active sites of different classes of these enzymes [18]. Quantum mechanical (QM) calculations demonstrated that the AdoMet methyl group forms relatively strong CH∙∙∙O hydrogen bonds due to its polarization by the neighboring sulfonium cation [18,19,20]. Correlatively, structural and biochemical characterization of the protein lysine N-methyltransferase (KMT) SET7/9 and the reactivation domain of methionine synthase demonstrated that these hydrogen bonds promote high affinity binding to AdoMet compared to the methyl transfer product S-adenosylhomocysteine (AdoHcy), thus mitigating product inhibition [18,21]. Moreover, CH∙∙∙O and CH∙∙∙N interactions with the AdoMet methyl group have been proposed to contribute to transition state stabilization in several methyltransferases, including SET7/9, SET8, NSD2, and glycine N-methyltransferase [18,22,23,24].

In addition to unconventional hydrogen bonding, chalcogen bonding between the AdoMet sulfur cation and the active sites of methyltransferases has also been observed [25]. A chalcogen bond is defined as a non-bonded interaction wherein a Lewis base donates a lone pair of electrons into the sigma antibonding (σ*) orbital of an atom from the Group VI elements (oxygen group) of the periodic table [26]. Structural and functional characterization of an S∙∙∙O chalcogen bond between AdoMet and an asparagine residue in the active site of SET7/9 demonstrated that this interaction enhances the binding affinity for the substrate relative to AdoHcy and modestly augments the rate of methyl transfer [25]. Together, these results illustrate that carbon hydrogen bonding and sulfur chalcogen bonding between the AdoMet sulfonium cation and residues in the methyltransferase active site can enhance the enzyme’s binding affinity for the substrate and promote the methyl transfer reaction.

Beyond hydrogen bonding and chalcogen bonding, there is a third unconventional interaction that can occur with sulfonium cations involving a σ* orbital of a carbon atom [27]. This interaction is termed a tetrel bond and occurs when an atom from the Group IV elements (carbon group) of the periodic table accepts a lone pair of electrons from an electronegative atom [28,29]. In the case of AdoMet, this interaction can occur with the σ* orbital of the methyl carbon atom that corresponds to the sulfur–carbon (S–CH_3_) bond.

Although aliphatic carbon atoms typically form weak tetrel bonds compared to other Group IV elements, QM calculations have demonstrated that a methyl carbon atom bonded to a sulfonium ion can form relatively strong tetrel interactions due to polarization by the adjacent cation [27]. Notably, the geometry of the tetrel bond, in which the interaction angle between the Lewis base (X) and S–CH_3_ bond is approximately linear, precludes strong methyl CH∙∙∙X hydrogen bonding due to acute hydrogen bond angles [27,30]. Experimental evidence for carbon tetrel interactions first emerged from a survey of the Cambridge Structural Database, which identified over 700 small molecule crystal structures displaying C∙∙∙O tetrel bonds, including multiple interactions involving methyl groups [31]. In addition, recent studies by Frontera and colleagues have revealed crystallographic evidence of methyl and trifluoromethyl C∙∙∙O tetrel bonding between proteins and various ligands [30,32]. Pertinent to methyltransferases, a computational analysis by Grabowski directly implicated tetrel bonding between an electrophilic tetrel atom and a nucleophile as a preliminary state that precedes the transition state in S_N_2 reactions, including methyl transfer [33]. Collectively, these findings prompted us to examine structures of AdoMet-dependent methyltransferases to ascertain whether methyl tetrel bonding occurs in these enzymes. The results of our structural survey coupled with corroborative QM calculations demonstrate the existence of the tetrel bonding in methyltransferases, furnishing insights into the potential roles of these interactions in ligand binding and S_N_2 catalysis.

## 2. Material and Methods

### 2.1. PDB Survey

Crystal structures of methyltransferase/AdoMet complexes with a resolution of ≤2.50 Å were downloaded from the PDB and visually examined for the presence of carbon tetrel bonding to the AdoMet methyl group. Tetrel bonds between the AdoMet methyl group and an electronegative atom (X) of a small molecule inhibitor, solvent molecule, or ion were defined as exhibiting: (1) an θ(S–C∙∙∙X) interaction angle between 160° and 180° (where S and C are the sulfur and methyl carbon atoms of AdoMet, respectively) and (2) a C∙∙∙X interaction distance less than or equal to sum of the van der Waals radii of the carbon and electronegative atoms, specifically R(C∙∙∙O) ≤3.25 Å and R(C∙∙∙Cl) ≤3.5 Å (carbon, oxygen, and chlorine van der Waals radii were defined as 1.75 Å, 1.5 Å, and 1.75 Å, respectively) [34]. These geometric parameters are consistent with the formal definition of halogen bonding, a related category of interactions that are considered an archetype for σ-hole bonding [35]. For crystal structures displaying potential carbon tetrel bonds, the electron density corresponding to AdoMet and the electron donor were visually inspected using the program Coot to confirm the integrity of the model [36,37]. Structures that displayed ambiguous electron density for the ligands were omitted from the survey. For the structure of the DhpI phosphonate *O*-methyltransferase (accession code 3OU6.pdb), the AdoMet molecules were remodeled in the electron density maps using the real space refinement and geometry tools in Coot. The remodeled AdoMet coordinates were then used to measure the tetrel bond geometries (Table 1). Finally, in cases where two or more structures of a given methyltransferase possess the same tetrel bond donor, such as interactions involving water molecules and the COMT/AdoMet/DNC/Mg^2+^ complexes, only the highest resolution structure of the wild type enzyme is reported in Table 1.

### 2.2. QM Calculations

Quantum calculations were carried out within the framework of the Gaussian-09 program. Active site models for SMYD2 (5ARG.pdb), SMYD3 (5CCL.pdb), COMT (5LSA.pdb), and G9A (5VSC.pdb) were generated from their respective crystallographic coordinates, with the heavy atom (non-hydrogen) positions fixed. Hydrogen atom positions were not derived from the enzymes’ X-ray structures but were added to the models followed by optimization of their positions at the M06-2X/6-31 + G** level. Energetics and NBO analyses [38,39] were performed at the M06-2X level with a larger aug-cc-pVDZ basis set. Interaction energies were evaluated as the difference in energy between the full system on one hand, and the sum of its components, as defined in the text, on the other. These quantities were corrected for the basis set superposition error with the counterpoise method. Spectral properties were computed at the M06-2X/6-31 + G** level. Interactions of each system with a polarizable medium were estimated via the CPCM method [40]. NMR data were computed with the GIAO approximation [41,42].

## 3. Results

### 3.1. Methyltransferase Structural Survey

A comprehensive survey of methyltransferase crystal structures (≤2.5 Å resolution) in the Protein Data Bank (PDB) was conducted to determine whether these enzymes exhibit evidence of methyl tetrel bonding. The survey comprised 269 structures and identified 20 nonredundant structures that display interaction geometries consistent with tetrel bonding between the AdoMet methyl group and ligands, ions, or solvent molecules within the active site (Table 1). Notably, no methyl tetrel bonding was observed between AdoMet and residues in the methyltransferases because the active sites of these enzymes preferentially orient the methyl group for nucleophilic attack by the methyl acceptor substrate. This finding explains the preponderance of AdoMet methyl tetrel bonding with ligands, ions, and solvent occupying the acceptor substrate binding cleft and thus the overall low percentage of methyltransferase structures displaying tetrel interactions. In contrast, CH∙∙∙O hydrogen bonding between the AdoMet methyl group and active site residues was observed in a high proportion of methyltransferase structures, as these interactions mediate substrate recognition by the enzymes and promote the alignment of the methyl group during the S_N_2 reaction [18,43].

The majority of the interactions observed in the PDB survey represent methyl C∙∙∙O tetrel bonds, with the exception of a single structure displaying a C∙∙∙Cl tetrel interaction between AdoMet and a small molecule inhibitor. The enzymes exhibiting tetrel bonding belong to either the (1) canonical class I methyltransferases (also known as the Rossmann fold-like or seven β-stranded methyltransferases) or (2) the Suppressor of variegation, Enhancer of Zeste, and Trithorax (SET) domain class of KMTs. This finding is not unexpected, given that these two classes are among the most abundant methyltransferases [2,3]. Furthermore, several members of the class I methyltransferases and SET domain KMTs are drug targets [44,45,46,47,48], resulting in the determination of multiple structures of these enzymes bound to various ligands.

**Figure 1:**
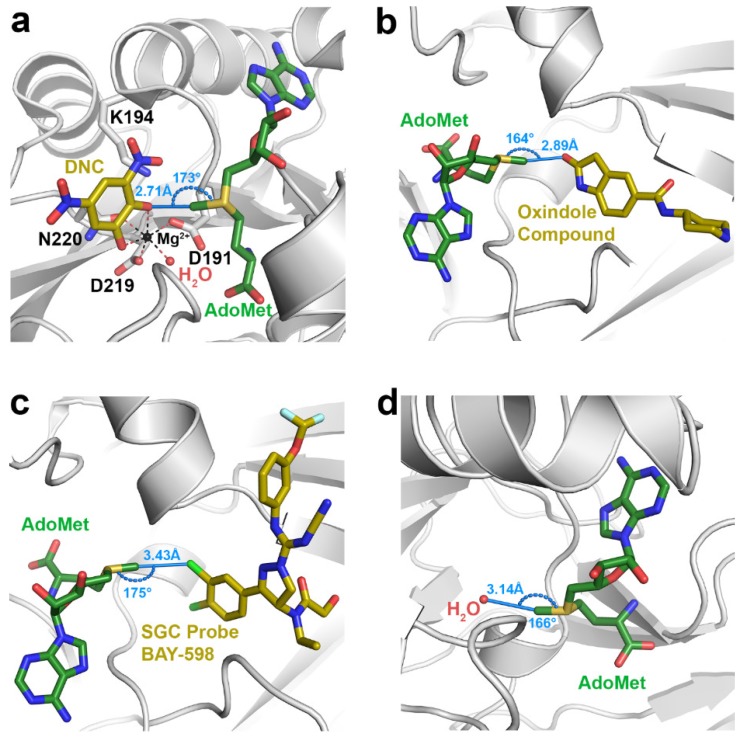
Representative examples of methyl tetrel bonding in crystal structures of AdoMet-dependent methyltransferases. AdoMet and small molecule inhibitors are depicted with green and yellow carbon atoms, respectively. Methyl tetrel bonding distances R(C∙∙∙X) and angles θ(S–C∙∙∙X) are denoted in blue. (**a**) COMT bound to AdoMet, DNC, and an Mg^2+^ ion (PDB accession code 5LSA). Key active site residues are illustrated, including the Mg^2+^-coordinating residues and the catalytic Lys194. (**b**) The SET domain KMT SMYD3 bound to AdoMet and an oxindole-containing inhibitor (5CCL). (**c**) SMYD2/AdoMet/SGC Probe BAY-598 ternary complex (5ARG). (**d**) SET domain KMT G9A bound to AdoMet and Inhibitor 13 (not shown) (5VSC). A water molecule in the substrate lysine binding channel of the enzyme engages in a methyl C∙∙∙O tetrel bond with AdoMet.

Among the class I methyltransferases, several inhibitor-bound structures of catechol *O*-methyltransferase (COMT) display interactions indicative of C∙∙∙O tetrel bonding (Table 1). COMT catalyzes the methylation of the hydroxyl groups of catechol substrates, such as norepinephrine, epinephrine, and dopamine, representing an initial step in their degradation [49]. Given its role in catechol catabolism, COMT represents an important drug target for treating neurological disorders such as Parkinson’s Disease and schizophrenia [45,49]. The COMT inhibitors identified in the survey represent catechol or catechol-like substrate analogs that mimic the binding of the substrate in the active site [46,50,51,52], as illustrated by the ternary complex of the enzyme bound to AdoMet and 3,5-dinitrocatechol (DNC) (Figure 1a). The interaction distances between the AdoMet methyl group and the oxygen atoms in the catechol analog inhibitors (R(C∙∙∙O) = 2.5–2.8 Å) are considerably shorter than the sum of the carbon and oxygen van der Waals radii (3.25 Å), indicative of strong tetrel bonding. Correlatively, Vidgren et al. noted a 2.6 Å C∙∙∙O interaction between the AdoMet methyl group and oxygen atom of DNC in the first crystal structure of COMT [53]. These short interaction distances are presumably a consequence of the protonation state of the catechol hydroxyl group participating in the tetrel bond. Catechol substrates and analog inhibitors have been posited to bind to COMT as a deprotonated catecholate due to stabilization of the phenoxide anion through resonance with the aromatic ring and its substituents, as well as by coordination to the Mg^2+^ cation in the active site [9]. The effect of the catecholate charge on AdoMet methyl C∙∙∙O tetrel bonding is investigated computationally in Section 3.2.

The SET domain KMTs represent the second methyltransferase class exhibiting evidence of AdoMet methyl tetrel bonding. These KMTs comprise several sub-classes, including the SET and MYND (Myeloid-Nervy-DEAF1) Domain-containing (SMYD) methyltransferases [54]. The human genome encodes five SMYD homologs, several of which have been implicated in cancer and cardiovascular disease, rendering them targets for drug design [54,55,56]. In particular, multiple structures of SMYD2 and SMYD3 in complex with various small molecule inhibitors have been determined [57,58,59]. Several of these structures display methyl C∙∙∙O tetrel bonding (Table 1), as illustrated in the ternary complex of SMYD3, AdoMet, and an oxindole-containing compound (Figure 1b). Notably, the SMYD2 and SMYD3 inhibitors that form methyl C∙∙∙O tetrel bonds with AdoMet are structurally dissimilar, unlike the catechol-based inhibitors of COMT. Unique among the structures in the survey, the SMYD3 inhibitor SGC Probe Bay-598 engages in an unusual C∙∙∙Cl tetrel bond with the methyl group of AdoMet (Figure 1c). The length of this tetrel interaction (3.43 Å) is longer than that observed in C∙∙∙O tetrel bonding due to the larger van der Waals radius of chlorine (Table 1). In summary, the inhibitor-bound structures of SMYD2 and SMYD3 illustrate that structurally diverse molecules can engage in AdoMet methyl tetrel bonding and that the electron donor is not limited to oxygen atoms, as halogens and potentially other Lewis bases can engage in tetrel interactions with the substrate.

In addition to interactions with small molecule inhibitors, the PDB survey also uncovered evidence of tetrel bonding between the AdoMet methyl group and solvent molecules as well as ions bound within methyltransferase active sites (Table 1). There are several structures that display C∙∙∙O tetrel bonding between AdoMet and water molecules, including the class I methyltransferases PrmA, PRMT5, and RsmF, as well as the SET domain KMTs ASH1L, MMSET, GLP, and G9A (Figure 1d). Similarly, the hydroxyl groups of ethylene glycol and glycerol engage in methyl C∙∙∙O tetrel bonding with AdoMet, as observed in the structures of SMYD2, SMYD3, and the class I enzyme Bud23 (Table 1). In addition to solvent molecules, methyl C∙∙∙O tetrel bonding is observed between AdoMet and a sulfate anion in the structure of the phosphonate *O*-methyltransferase DhpI. The sulfate anion bound in the enzyme’s active site has been proposed to mimic the phosphonate group of the methyl acceptor substrate [60], suggesting that the sulfate may function as a non-reactive substrate analog, similar to the catechol-based inhibitors of COMT. Consistent with this observation, the C∙∙∙O tetrel bonds between AdoMet and sulfate are generally shorter and closer to linearity than the tetrel interactions involving solvent molecules (Table 1). Thus, these interactions may potentially represent a Michaelis complex-like state in DhpI and mimic the reaction coordinate for phosphonate methylation. Finally, the finding that AdoMet methyl tetrel bonds involving solvent molecules tend to be longer (R(C∙∙∙O) = 3.0–3.25 Å) than the interactions observed in the complexes with inhibitors and substrate analogs (Table 1) implies that the solvent interactions may be energetically weaker. This observation is examined in Section 3.2.

### 3.2. Computational Results

**Figure 2:**
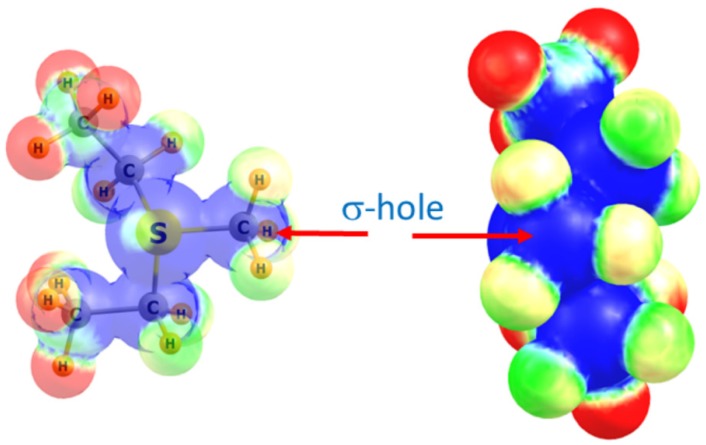
Two views of the molecular electrostatic potential surrounding the MeS^+^(Et)_2_ sulfonium cation. Right view looks directly down the H_3_C–S axis. Blue and red colors correspond respectively to +0.40 and +0.30 au.

After completing the PBD survey, we selected four methyltransferase structures for computational analysis to investigate the theoretical energies and spectroscopic properties of the observed tetrel bonds with the AdoMet methyl group. These structures include the COMT/AdoMet/DNC/Mg^2+^, SMYD3/AdoMet/oxindole, and SMYD2/AdoMet/SGC BAY-598 complexes, as well as the G9A structure exhibiting a C∙∙∙O tetrel bond between AdoMet and a water molecule (Figure 1). For the purposes of the QM calculations, AdoMet was represented as the sulfonium cation MeS^+^(Et)_2_, as previously reported [21,25,61]. The ability of the methyl group of this moiety to engage in a tetrel bond was first examined by computing its molecular electrostatic potential (MEP), as illustrated in Figure 2. As a cation, the MEP is positive at all positions with the most positive regions highlighted in blue. There is a region of blue along the extension of the S–CH_3_ bond, corresponding to the σ* orbital that is also referred to as a σ-hole. A maximum occurs on the isodensity surface (0.05 au) with a value of +120 kcal/mol. It is in this region of the surface that Coulombic attraction with a Lewis base may occur.

**Figure 3:**
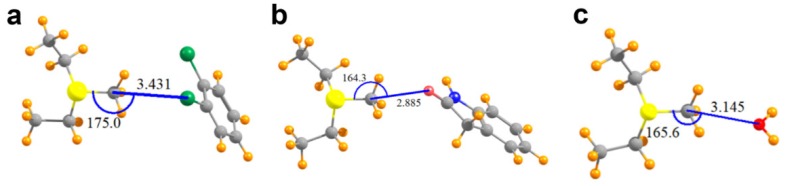
Molecular structures of the models used to computationally analyze AdoMet methyl tetrel bonding in SET domain KMTs. (**a**) Model of MeS^+^(Et)_2_ and dichlorobenzene in the SMYD2 structure; (**b**) MeS^+^(Et)_2_ and oxindole in the SMYD3 structure; (**c**) MeS^+^(Et)_2_ and a water molecule bound in the active site of G9A. Values for the tetrel bond distances and angles are reported in Angstrom and degrees, respectively.

**Figure 4:**
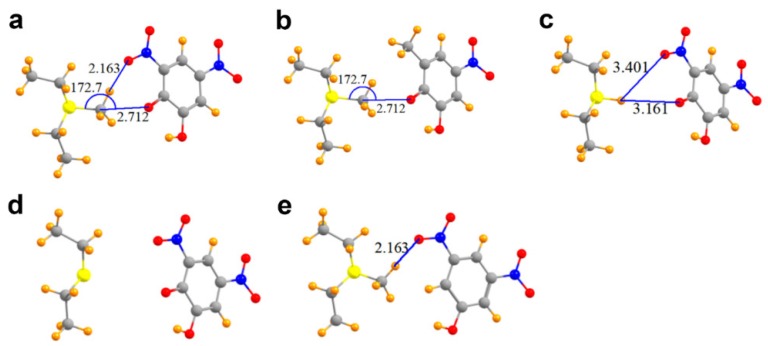
Molecular structures of COMT models used to probe C∙∙∙O tetrel bonding between AdoMet and DNC. (**a**) MeS^+^(Et)_2_ and the phenoxide anion of DNC; (**b**) Model in which the 3-nitro moiety of DNC is replaced with a methyl group; (**c**) Complex wherein the methyl group of MeS^+^(Et)_2_ is replaced with a hydrogen atom, yielding HS^+^(Et)_2_; (**d**) Model of DNC and the thioether S(Et)_2_ representing the product AdoHcy; (**e**) Complex in which the phenoxide oxygen atom of DNC is substituted by a hydrogen atom.

The geometries of the pair of relevant interacting groups in the structures of SMYD2, SMYD3, G9A, and COMT are depicted in Figure 3 and Figure 4a. The Lewis base in SMYD2 is represented by the o-dichlorobenzene group of the SGC BAY-598 compound, whereas the oxygen electron donor in the SMYD3 complex is represented by the oxindole moiety of the inhibitor. In G9A, a water molecule serves as the Lewis base. In the COMT model, the oxygen electron donor was modeled as a DNC phenoxide anion. The pKa value for the methyl-interacting hydroxyl group is estimated to be ~3.3 and thus has been predicted to bind to the enzyme in a deprotonated state [9]. Together, these four systems cover a range of attributes of potential tetrel bonds. The SMYD2 complex involves a Cl atom as the Lewis base, whereas the more typical oxygen atom assumes this role in the SMYD3, G9A, and COMT complexes. While the first three systems pair the MeS^+^(Et)_2_ cation with a neutral partner, the COMT model contains a formal negative charge from the phenoxide anion of DNC.

As illustrated in Table 2, the putative tetrel bond is longest in SMYD2 with an intermolecular R(C∙∙∙Cl) distance of 3.43 Å, and shortest in COMT with R(C∙∙∙O) = 2.71 Å. All are reasonably close to linearity, the least of which is the θ(S–C∙∙∙O) angle of 164° in SMYD3/AdoMet/oxindole complex. Table 2 also reports the interaction energies between the two monomers as E_int_, where a negative quantity indicates an attractive interaction. As a frame of reference, an O–H∙∙∙O hydrogen bond in a water dimer has an interaction energy of −5.8 kcal/mol when calculated at this level of QM theory [18]. The SMYD2/AdoMet/SGC Probe BAY-598 complex containing the longest of the tetrel bonds, with R(C∙∙∙Cl) = 3.43 Å, is bound by −5.2 kcal/mol. The shorter bond of 2.89 Å in SMYD3 is associated with nearly twice the interaction energy, despite the 10° loss of linearity. Even a C∙∙∙O length exceeding 3.14 Å in the G9A/AdoMet/H_2_O complex is associated with a substantial bond energy of −7.0 kcal/mol. A much larger increment, raising the bonding energy to −65.7 kcal/mol, occurs when the partner subunit is negatively charged.

In order to probe the nature of the interaction, the wave function was analyzed by the NBO procedure which considers charge transfers from one molecular orbital to another. E^T^ represents the perturbation energy consequence of transfer from the Lewis base (X) lone pair to the σ*(C–S) antibonding orbital, indicative of tetrel bond formation. Because of the proximity of CH protons to the nucleophile, there is the alternate possibility of a CH∙∙∙X hydrogen bond, which would manifest itself by a transfer into the σ*(H–C) antibonding orbital. Such a possibility is measured by E^H^, which is reported in the last column of Table 2. A glance at the last two columns makes it clear that, while there may be a small amount of hydrogen bonding, particularly in the SMYD3 complex with the least linear θ(S–C∙∙∙O) angle, the interaction is nonetheless dominated by E^T^ and tetrel bonding. Most importantly, this tetrel bonding is quite strong, as much as −9 kcal/mol for the neutral nucleophile, rising to more than −60 kcal/mol when the latter is an anion.

An additional means to establish the presence of a tetrel bond, which can also distinguish this sort of interaction from a CH∙∙∙X hydrogen bond is by means of NMR and IR spectral data. A recent study [62] computed these quantities for a range of different complexes in which a methyl group is situated close to a nucleophile, in arrangements much like those considered here. In the case where a tetrel bond is unequivocally present, the chemical shielding of the methyl carbon nucleus is reduced by some 2–14 ppm, relative to the uncomplexed Lewis acid, depending upon the particular system. The methyl protons are deshielded as well, but by much smaller amounts, generally less than 1 ppm. This pattern effectively reverses in the case of a CH∙∙∙X hydrogen bond where it is the methyl protons that are more strongly deshielded than the carbon nucleus. The vibrational frequencies of the methyl group can also be used to characterize the presence of a tetrel or hydrogen bond. Most diagnostic are the symmetric stretch and bend. The former undergoes a small blue shift in a tetrel bond, but a much larger red shift when it is a hydrogen bond that is present. The symmetric bend, or umbrella mode, is strongly red-shifted for a tetrel bond, but turns toward a blue shift for a hydrogen bond.

With these patterns in mind, Table 3 provides further confirmation of the tetrel bonds that are present in these systems. The methyl carbon atom is deshielded by between 2 and 6 ppm, an amount much larger in magnitude than the deshielding of the methyl protons, less than 1 ppm. The symmetric stretching frequency rises by a small amount, and the umbrella bend is very substantially red-shifted. All of these trends fit perfectly into the aforementioned spectroscopic fingerprint of a tetrel bond. Note also that the quantitative values of these changes follow the same order as do the interaction energies of the three systems listed in Table 2, with the largest changes associated with the COMT complex.

A glance at Figure 4a suggests the likelihood of a hydrogen bond connecting one of the three methyl CH protons with a nitro oxygen atom, since these two atoms lie only 2.16 Å apart. In addition, E for this secondary interaction amounts to −6.29 kcal/mol, larger than that for the tetrel bond itself (this quantity is not reported in Table 2 as it refers to a separate interaction that does not involve the methyl carbon atom directly). Moreover, the electron density at the AIM bond critical points, generally considered an accurate barometer of noncovalent bond strength, are 0.015 and 0.017 au for the C∙∙∙O tetrel bond and CH∙∙∙O hydrogen bond, respectively.

These two seemingly similar attractive forces need to be disentangled so as to better estimate the interaction energy of the tetrel bond itself. One way to evaluate the latter quantity is to remove the hydrogen bond entirely. Replacement of the nitro (NO_2_) moiety by a simple methyl group deletes any possible hydrogen bond, while leaving the tetrel bond intact. The interaction energy of the resulting derivative dimer in Figure 4b is −62.1 kcal/mol, less attractive than the full complex by 3.6 kcal/mol, providing one estimate of the hydrogen bond energy. The replacement of the nitro moiety by a methyl group has secondary effects in that, for example, the removal of the electron-withdrawing nitro group would tend to make the phenoxide oxygen atom a bit more potent Lewis base, which would in turn amplify the interaction energy. One can effectively eliminate both the tetrel and hydrogen bonds by replacing the methyl group by a hydrogen atom. This hydrogen atom in Figure 4c is too far away from either the phenoxide oxygen (3.16 Å) or the nitro oxygen atom (3.40 Å) to engage in any substantive bond. The interaction energy in this case is reduced to −60.5 kcal/mol, 5.2 kcal/mol less than the full system, which includes both sorts of bonds. Much of the remaining attractive force resides in the simple cation∙∙∙anion Coulombic ion pair interaction. If the methyl group is removed from MeS^+^(Et)_2_, leaving behind a neutral S(Et)_2_ (representing the product AdoHcy) in Figure 4d, while also deleting both the tetrel and hydrogen bond, the interaction energy is reduced to 0.

Still another scheme to dissect the total interaction into its component segments arises if the phenoxide oxygen atom of DNC is replaced by a hydrogen atom, which would eliminate the tetrel bond, while retaining the CH∙∙∙O hydrogen bond in Figure 4e. If this exchange occurs while retaining the negative charge of the DNC (a doublet), the interaction energy is −58.4 kcal/mol. This quantity is some 7.3 kcal/mol less attractive than that with the phenoxide oxygen, providing an estimate of the tetrel bond energy. On the other hand, if the modified DNC is made electrically neutral (a singlet), the interaction energy is reduced to zero. In other words, in the absence of the strong Coulombic ion pair interaction, any CH∙∙∙O hydrogen bond in this system is quite weak despite the close R(H∙∙∙O) distance of 2.16 Å.

**Figure 5:**
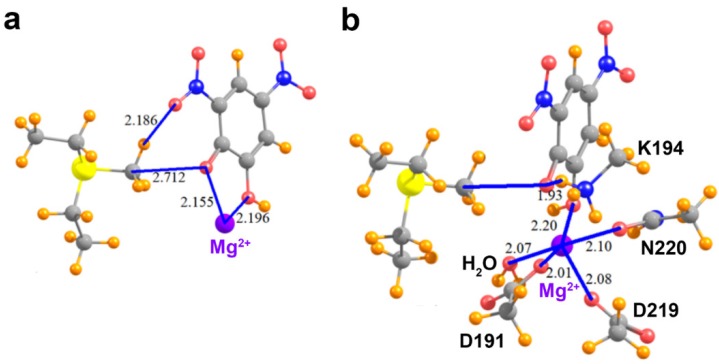
Molecular structures of COMT active site models. (**a**) MeS^+^(Et)_2_, the phenoxide anion of DNC, and the active site Mg^2+^ ion; (**b**) The model depicted in (**a**) that also includes the residues and water molecule that coordinate the Mg^2+^ ion and the catalytic residue Lys194.

We next consider the effects of other residues on the foregoing analysis. Unlike the KMTs G9A, SMYD2, and SMYD3, COMT possesses an active site Mg^2+^ ion that promotes the deprotonation of the catechol substrate’s reactive hydroxyl group, forming the phenoxide anion through metal ion catalysis [49]. Notably, the phenoxide anion of DNC directly coordinates to this metal ion (Figure 5a). Thus, additional models were generated to examine the effect of the Mg^2+^ ion on the methyl C∙∙∙O tetrel bond between AdoMet and DNC. In the model that only adds the Mg^2+^ ion (Figure 5a), its divalent charge acts to repel the MeS^+^(Et)_2_ cation, such that the interaction of these two species, without the DNC, amounts to 111.2 kcal/mol. The interaction energy of the MeS^+^(Et)_2_ with the DNC∙∙∙Mg^2+^ pair cannot overcome this strong repulsive force, so is +48.2 kcal/mol. When the pure MeS^+^(Et)_2_∙∙∙Mg^2+^ repulsion is subtracted from this quantity, one finds that the interaction between MeS^+^(Et)_2_ and DNC is attractive, in the amount of −63.0 kcal/mol. This quantity differs from the −65.7 kcal/mol pure MeS^+^(Et)_2_∙∙∙DNC interaction, in the complete absence of Mg^2+^ (see Table 2) by 2.7 kcal/mol. In other words, the presence of the divalent cation reduces the tetrel/hydrogen bond energy by only 4%.

As the Mg^2+^ ion is coordinated by several residues in COMT, it is important to assess how the metal coordination affects tetrel bonding between AdoMet and DNC. Within the enzyme’s active site, the side chains of Asp191, Asp219, and Asn220 coordinate to Mg^2+^ with the last coordination site occupied by a water molecule. To represent these interactions, the aspartate and asparagine side chains were modeled as acetate and acetamide, respectively, and the water molecule was included to complete the metal’s coordination sphere (Figure 5b). In addition, this model included the catalytic base, Lys194, that deprotonates the reactive hydroxyl group of the catechol substrate [49]. The lysine side chain was represented by a methyl ammonium cation and forms an NH∙∙∙O hydrogen bond with the phenoxide anion of DNC, mimicking the deprotonation of the catechol hydroxyl group prior to methyl transfer. The presence of the Asp191 and Asp219 carboxylate anions tempers the effects of the Mg^2+^ to some degree. The interaction energy of the MeS^+^(Et)_2_ with the new system elements, i.e., all exclusive of the DNC, is +47.4 kcal/mol. The total interaction when the DNC is added to the entire system is −16.3 kcal/mol. After subtraction of the repulsion between the Lewis acid and the extraneous elements, the interaction energy of the MeS^+^(Et)_2_ with DNC is −63.7 kcal/mol. This quantity represents a very small decrease relative to the naked MeS^+^(Et)_2_/DNC dimer, in the amount of 3%. In summary, the interaction energy computed for a pair of species, interacting directly via a combined tetrel and hydrogen bond, is scarcely affected by the addition of surrounding groups, provided one takes proper account of the newly introduced pairwise interactions.

It is worth noting that the calculations above placed each system into an in vacuo situation, removed from the electrostatic or polarizing influence of neighboring groups. It is anticipated that these external effects will ameliorate some of the strongest Coulombic forces between the two groups, at least one of which carries a full charge. In an effort to estimate how much the surrounding protein environment might weaken these charge-assisted interactions [63], the various systems listed in Table 2 were placed within a polarizable continuum of dielectric constant ε = 4, a value that is commonly taken as the average value within a protein interior and that has been used several times [64,65,66] to good effect. Indeed, a weakening was observed, reducing the interaction energies by a factor between two and three. Nonetheless, the interactions remain strong, as large as −19.2 kcal/mol for the COMT system.

## 4. Discussion

As in the case of halogen, chalcogen, and pnictogen bonds, tetrel bonds are much stronger for elements in lower rows of the periodic table, e.g., Si and Ge. For this reason, the majority of computational work [67,68,69,70,71,72,73,74,75,76,77] has been dedicated to these stronger interactions. However, it is to the nominally weaker carbon tetrel bonds that this work is devoted, due in part to their prevalence in biological systems. While this survey and calculations have focused specifically on AdoMet-dependent methyltransferases, there is ample evidence that carbon tetrel bonds occur in a host of other systems. Thorough reviews of a variety of structures yield numerous interactions, on the order of thousands, where a powerful Lewis base is poised in the precise position, near to the extension of the R–C bond axis, that is consistent with a tetrel bond [32,78,79]. In the specific case of methyl groups, Guru Row and coworkers [31] identified more than 700 structures in the CSD where the interaction fits the geometrical requirements, and provided confirmation based on analyses of experimental charge density. In general, methyl groups are capable of forming only weak tetrel bonds [80] without the amplification that arises from either the presence of electron-withdrawing substituents, a strong base, charge assistance in the form of either a cationic Lewis acid or anionic base, or cooperative effects [33,74,81,82,83,84,85,86,87,88,89,90,91]. For example, the tetrel bond between S(CH_3_)_3_^+^ and N-methylacetamide amounts to −13.7 kcal/mol, but to only −1.9 kcal/mol for the uncharged analogue S(CH_3_)_2_ [27].

Our finding here that the interaction can be better described as a tetrel bond than as a trifurcated CH∙∙∙O hydrogen bond to a methyl group is consistent with earlier calculations [30]. This group has also performed calculations that confirm the presence of tetrel bonds in selected structures from the PDB [32], although that work was limited to the highly substituted CF_3_ rather than pure methyl groups. Nonetheless, clear evidence was presented for the presence of tetrel bonds to this sp^3^-hybridized carbon atom.

The discovery of carbon tetrel bonding in AdoMet-dependent methyltransferases has important ramifications with respect to our understanding of the catalytic mechanism of these enzymes. As illustrated in prior computational studies, tetrel bonding between the electrophile and nucleophile represents a preliminary state that precedes the transition state in methyl transfer and other S_N_2 reactions [33]. Much of our present knowledge of S_N_2 catalysis in methyltransferases is derived from decades of paradigmatic studies of COMT and has resulted in several models for the reaction mechanism of these enzymes. Serendipitously, crystal structures of COMT bound to AdoMet and substrate analog inhibitors, such as DNC, have provided strong evidence for C∙∙∙O tetrel bonding, given the close interaction distances of the AdoMet methyl group and phenoxide anion of DNC, as well as the S–C∙∙∙O interaction angle that approaches linearity (Table 1 and Figure 1a). Thus, the tetrel interaction not only establishes the prerequisite geometry for the S_N_2 reaction, but also aligns the lone pair of electrons of the nucleophile with the σ* orbital of the AdoMet methyl carbon atom. This orbital alignment promotes the formation of the bond between the methyl carbon and the nucleophile in the transition state.

The identification of carbon tetrel bonding also furnishes experimental explanations for certain models of the methyltransferase catalytic mechanism that are based on studies of COMT and other enzymes. The compression/compaction model postulates that the methyltransferase active site essentially squeezes the distances along the S∙∙∙CH_3_∙∙∙X reaction coordinate in the transition state (where X is the nucleophile) [6,7]. As observed in the COMT/AdoMet/DNC/Mg^2+^ complex (Figure 1a), the C∙∙∙O tetrel bond between the AdoMet methyl group and phenoxide anion of DNC, representing a catechol substrate, is 2.71 Å, 0.54 Å shorter than the carbon–oxygen van der Waals contact distance of 3.25 Å. This short interaction distance is illustrative of the close approach of the two substrates postulated in the compression model. Importantly, the C∙∙∙O tetrel interaction is attractive in nature (Table 2) and would result in an electron charge transfer from the nucleophile to the methyl carbon atom. In turn, this transfer would polarize and weaken the S–CH_3_ bond during the S_N_2 reaction [33]. The presence of methyl tetrel bonds observed in COMT also concurs with the NAC model for the catalytic mechanism of COMT and other methyltransferases. In molecular dynamic simulations, the NACs that transiently formed between AdoMet and a catechol bound to COMT were defined as having a C∙∙∙O interaction distance of <3.2 Å and an S–C∙∙∙O interaction angle ≥165° [12,92]. This geometry mirrors the interaction distances and angles observed for tetrel bonding (Figure 1 and Table 1). It is conceivable that the favorable energy of C∙∙∙O tetrel bonding would increase the frequency in which AdoMet and the catechol substrate are brought into a catalytically productive alignment that is conducive to methyl transfer.

Knowledge of carbon tetrel bonding can also be leveraged in the design of methyltransferase inhibitors. Indeed, the PDB survey illustrates several examples of inhibitors of COMT and SET domain KMTs that form tetrel bonds with the AdoMet methyl group (Table 1 and Figure 1). In the case of catechol analog inhibitors of COMT, the addition of nitro groups or other electron withdrawing moieties to the catechol ring (such as in DNC), or substitution of the catechol phenyl ring with pyridine or other six-membered heterocycles diminishes the nucleophilicity of the reactive hydroxyl group, abrogating methyl transfer with AdoMet. It has been proposed that these inhibitors bind to the COMT and AdoMet as a deprotonated phenoxide anion [9], which can engage in strong methyl C∙∙∙O tetrel bonding with AdoMet. The calculated interaction energy of the C∙∙∙O tetrel interaction between the DNC phenoxide anion and the AdoMet methyl group is substantially stronger than tetrel interactions involving neutral oxygen atoms in carbonyl and hydroxyl groups (Table 2). In agreement with these findings, nitrocatechol inhibitors of COMT, such as DNC, tolcapone, and entacapone, display equilibrium inhibitory constants (*K_I_*) that are in the low nanomolar to picomolar range [93,94]. The tight binding of these inhibitors to the enzyme may be mediated in part by the strong C∙∙∙O tetrel bonding between the phenoxide anion and AdoMet methyl group.

In addition to COMT, inhibitors of the SMYD KMTs display methyl tetrel bonding with AdoMet (Table 1). With respect to SMYD2, structure–activity relationship (SAR) analysis of the BAY-598 inhibitor revealed a preference for a chlorine atom in the 3-position of the phenyl moiety [58]. This chlorine atom corresponds to the Lewis base that forms the C∙∙∙Cl tetrel bond with AdoMet (Figure 1c). Kinetic analysis of BAY-598 demonstrated uncompetitive inhibition toward AdoMet, indicating the inhibitor binds exclusively to the SMYD2/AdoMet binary complex and thus does not recognize the free enzyme. It is conceivable that the C∙∙∙Cl tetrel bond between AdoMet and SGC Probe Bay-598 in SMYD2 may contribute to this uncompetitive inhibition by promoting recognition of the inhibitor by the enzyme/substrate complex. Through compound screening and SAR analysis, Mitchell and coworkers identified an oxindole class of inhibitors that selectively inhibits SMYD3 [57]. Crystallographic characterization of the initial oxindole hit (Figure 1b) and the SAR-optimized inhibitor EPZ030456 bound to SMYD3 and AdoMet revealed methyl C∙∙∙O tetrel bonding with the oxygen atom of the oxindole moiety (Table 1). The structural conservation of the methyl C∙∙∙O tetrel bond formed by the oxindole inhibitors suggests that this interaction may be important for recognition and selectivity for SMYD3.

## 5. Conclusions

The survey of AdoMet-bound methyltransferase structures in the PDB uncovered a number of geometries that strongly indicate the presence of methyl tetrel bonding to electronegative atoms in small molecule inhibitors, solvent molecules, and ions. The C∙∙∙X distances between the AdoMet methyl carbon atom and the Lewis base vary over a wide range, but all of the C∙∙∙O interactions reported from this survey are less than 3.25 Å, with some distances as short as 2.5 Å. Further, the Lewis base is located close to the extension of the S–CH_3_ bond of AdoMet, with θ(S–C∙∙∙O) angles within 20° of linearity. QM analysis of a selected set of these interactions revealed unequivocal evidence of methyl tetrel bonds, rather than what might naively be considered a trifurcated CH∙∙∙O hydrogen bond. The interaction energies of these selected tetrel bonds varied between −5 and −9 kcal/mol, comparable to or stronger than the paradigmatic hydrogen bond between a pair of water molecules (−5.8 kcal/mol). In the case of a Lewis base bearing a full negative charge, the tetrel bond energy was amplified to more than −60 kcal/mol. It is thus clear that our understanding of the forces present within biological systems must include tetrel bonding on the same footing as the venerable hydrogen bond. Finally, the discovery of AdoMet methyl tetrel bonding in methyltransferases illustrates that this interaction may have a fundamental role in the catalytic mechanism of these enzymes and thus merits further investigation. An understanding of this novel interaction can be applied in structure-guided design of potent inhibitors of methyltransferases implicated in disease.

## Figures and Tables

**Table 1:** Crystallographic survey of methyl tetrel bonding in AdoMet-dependent methyltransferases.

Enzyme	PDB Code	Resolution (Å)	Ligand	Electron Donor (X)	R(C∙∙∙X) Length (Å) ^A^	θ(S–C∙∙∙X) Angle (°) ^A^
ASH1L	4YNM	2.19	H_2_O	O	2.99 (B)	163 (B)
Bud23	4QTU	2.12	Ethylene glycol	O	3.04 (B)	173 (B)
COMT	2CL5	1.6	BIA 8-176 ^B^	O	2.70 (A), 2.69 (B)	173 (A), 172 (B)
COMT	3S68	1.85	Tolcapone ^C^	O	2.50	166
COMT	4XUC	1.8	Compound 18 ^D^	O	2.64	175
COMT	4XUD	2.4	Compound 32 ^E^	O	2.73	166
COMT	5LSA	1.5	3,5-Dinitrocatechol	O	2.71	173
DhpI	3OU6	2.3	Sulfate	O	3.00 (A), 3.09 (B), 2.97 (C)	175 (A), 175 (B), 176 (C)
G9A	5VSC	1.4	H_2_O	O	3.14 (A), 3.17 (B)	166 (A), 168 (B)
GLP	5TTG	1.66	H_2_O	O	3.15 (A), 3.24 (B)	168 (A), 169 (B)
MMSET	5LSU	2.14	H_2_O	O	3.13 (B)	160 (B)
PrmA	2NXE	1.75	H_2_O	O	3.08 (B)	171 (B)
PRMT5	5EML	2.39	H_2_O	O	3.09 (A)	163 (A)
RsmF	3M6V	1.82	H_2_O	O	3.20 (A), 3.23 (B)	164 (A), 162 (B)
SMYD2	3S7B	2.42	AZ505 ^F^	O	2.77	169
SMYD2	3TG4	2.0	Glycerol	O	3.23	176
SMYD2	5ARG	1.99	SGC Probe BAY-598 ^G^	Cl	3.43	175
SMYD3	3QWP	1.53	Glycerol	O	3.01	163
SMYD3	5CCL	1.5	Oxindole compound ^H^	O	2.89	164
SMYD3	5CCM	2.3	EPZ030456 ^I^	O	2.78	168

Note: ^A^: A, B, and C denote the protein chains in the asymmetric unit of the crystal structure; ^B^: (3,4-dihydroxy-2-nitrophenyl)(phenyl)methanone; ^C^: (3,4-dihydroxy-5-nitrophenyl)(4-methylphenyl)methanone; ^D^: 1-(biphenyl-3-yl)-3-hydroxypyridin-4(1H)-one; ^E^: [1-(biphenyl-3-yl)-5-hydroxy-4-oxo-1,4-dihydropyridin-3-yl]boronic acid; ^F^: N-cyclohexyl-N~3~-[2-(3,4-dichlorophenyl)ethyl]-N-(2-{[2-(5-hydroxy-3-oxo-3,4-dihydro-2H-1,4-benzoxazin-8-yl)ethyl]amino}ethyl)-beta-alaninamide; ^G^: N-[1-(N’-cyano-N-[3-(difluoromethoxy)phenyl]carbamimidoyl)-3-(3,4-dichlorophenyl)-4,5-dihydro-1H-pyrazol-4-yl]-N-ethyl-2-hydroxyacetamide; ^H^: 2-oxidanylidene-N-piperidin-4-yl-1,3-dihydroindole-5-carboxamide; ^I^: 6-chloranyl-2-oxidanylidene-N-[(1S,5R)-8-[4-[(phenylmethyl)amino]piperidin-1-yl]sulfonyl-8-azabicyclo[3.2.1]octan-3-yl]-1,3-dihydroindole-5-carboxamide.

**Table 2:** Properties of tetrel bond in indicated systems, where X indicates nature of electron donor atom. Energetics are reported in kcal/mol.

Structure	PDB	X	R(C∙∙∙X) (Å)	θ(S–C∙∙∙X) (°)	E_int_	E^T^	E^H^
SMYD2	5ARG	Cl	3.431	175.0	−5.2	0.63	0.10
SMYD3	5CCL	O	2.885	164.3	−9.0	0.62	0.38
G9A	5VSC	O	3.145	165.6	−7.0	0.46	0.16
COMT	5LSA	O^-^	2.712	172.7	−65.7	1.33	0.16

E^T^: X_lp_ → σ*(SC) E^H^: X_lp_ → σ*(CH).

**Table 3:** Calculated changes in the NMR chemical shift (∆σ, ppm) and the symmetric stretching and bending frequencies (∆ν, cm^−1^) within the methyl group of MeS^+^(Et)_2_ caused by complexation.

Structure	PDB	∆σ_C_	∆σ_H_	∆ν_str_	∆ν_bend_
SMYD2	5ARG	−1.94	−0.14	0.8	−11.5
SMYD3	5CCL	−2.27	−0.28	5.7	−38.3
G9A	5VSC	−2.36	−0.29	3.9	−22.9
COMT	5LSA	−6.29	−0.95	4.8	−56.8
